# The Anti-Inflammatory Effect of Algae-Derived Lipid Extracts on Lipopolysaccharide (LPS)-Stimulated Human THP-1 Macrophages

**DOI:** 10.3390/md13085402

**Published:** 2015-08-20

**Authors:** Ruairi C. Robertson, Freddy Guihéneuf, Bojlul Bahar, Matthias Schmid, Dagmar B. Stengel, Gerald F. Fitzgerald, R. Paul Ross, Catherine Stanton

**Affiliations:** 1 Biosciences Department, Teagasc Food Research Centre, Moorepark, Fermoy, Co. Cork, Ireland; E-Mail: ruairi.robertson@teagasc.ie; 2 School of Microbiology, University College Cork, Co. Cork, Ireland; E-Mail: g.fitzgerald@ucc.ie; 3 Botany and Plant Science, School of Natural Sciences, Ryan Institute for Environmental, Marine and Energy Research, National University of Ireland Galway, Galway, Ireland; E-Mails: freddy.guiheneuf@nuigalway.ie (F.G.); M.SCHMID2@nuigalway.ie (M.S.); dagmar.stengel@nuigalway.ie (D.B.S.); 4 School of Agriculture and Food Science, Institute of Food & Health, University College Dublin, Belfield, Dublin 4, Ireland; E-Mail: bojlul.bahar@ucd.ie; 5 APC Microbiome Institute, University College Cork, Co. Cork, Ireland; E-Mail: p.ross@ucc.ie; 6 College of Science, Engineering and Food Science, University College Cork, Co. Cork, Ireland

**Keywords:** microalgae, macroalgae, THP-1, inflammation, lipids, *n*-3 PUFA, polyunsaturated fatty acids, macrophages, chlorophyll *a*, bioactive pigments

## Abstract

Algae contain a number of anti-inflammatory bioactive compounds such as omega-3 polyunsaturated fatty acids (*n*-3 PUFA) and chlorophyll *a*, hence as dietary ingredients, their extracts may be effective in chronic inflammation-linked metabolic diseases such as cardiovascular disease. In this study, anti-inflammatory potential of lipid extracts from three red seaweeds (*Porphyra dioica*, *Palmaria palmata* and *Chondrus crispus*) and one microalga (*Pavlova lutheri*) were assessed in lipopolysaccharide (LPS)-stimulated human THP-1 macrophages. Extracts contained 34%–42% total fatty acids as *n*-3 PUFA and 5%–7% crude extract as pigments, including chlorophyll *a*, β-carotene and fucoxanthin. Pretreatment of the THP-1 cells with lipid extract from *P. palmata* inhibited production of the pro-inflammatory cytokines interleukin (IL)-6 (*p* < 0.05) and IL-8 (*p* < 0.05) while that of *P. lutheri* inhibited IL-6 (*p* < 0.01) production. Quantitative gene expression analysis of a panel of 92 genes linked to inflammatory signaling pathway revealed down-regulation of the expression of 14 pro-inflammatory genes (*TLR1*, *TLR2*, *TLR4*, *TLR8*, *TRAF5*, *TRAF6*, *TNFSF18*, *IL6R*, *IL23*, *CCR1*, *CCR4*, *CCL17*, *STAT3*, *MAP3K1*) by the lipid extracts. The lipid extracts effectively inhibited the LPS-induced pro-inflammatory signaling pathways mediated via toll-like receptors, chemokines and nuclear factor kappa-light-chain-enhancer of activated B cells (NF-κB) signaling molecules. These results suggest that lipid extracts from *P. lutheri*, *P. palmata*, *P. dioica* and *C. crispus* can inhibit LPS-induced inflammatory pathways in human macrophages. Therefore, algal lipid extracts should be further explored as anti-inflammatory ingredients for chronic inflammation-linked metabolic diseases.

## 1. Introduction

Inflammation is a complex physiological process involving activation of the immune system. It occurs following physical injury or invasion by pathogenic bacteria, viruses or tumor cells in the host [1]. Inflammation is essential to identify and destroy invading pathogens and is vital for host health in acute disease states. However, in many cases, inflammation occurs at a chronic, subclinical level and if this process becomes unregulated and prolonged, the activated immune system can begin to damage host tissues and supplement chronic disease states such as cardiovascular disease (CVD) [2], inflammatory bowel disease (IBD) [3], obesity [4,5] and Alzheimer’s disease (AD) [6]. Dietary interventions to control chronic inflammation may provide useful strategies for the management of such diseases [7,8,9,10], therefore the discovery of novel, effective, anti-inflammatory bioactives is warranted to identify long-term dietary therapies for inflammatory metabolic disorders.

The use of synthetic pharmacological agents to inhibit inflammation has shown promise in metabolic diseases such as type 2 diabetes and CVD [11]; however, chronic use of such drugs is often associated with adverse gastrointestinal side effects [12]. The application of natural anti-inflammatory compounds is desirable as a long-term strategy to suppress chronic inflammation and alleviate disease-associated symptoms. A number of dietary components have been shown to exhibit potent anti-inflammatory activity, and evidence suggests certain dietary changes may be key as long-term alternatives to pharmaceutical compounds [8]. Long-chain *n*-3 polyunsaturated fatty acids (*n*-3 LC-PUFA), for example, have been demonstrated to reduce inflammation in both *in vitro* and *in vivo* studies [1,13,14,15]. *n*-3 LC-PUFA act as precursors to potent anti-inflammatory mediators, termed eicosanoids, which inhibit inflammatory cytokine production and inflammatory gene expression [16]. Indeed, the *n*-3 LC-PUFA eicosapentaenoic acid (EPA, 20:5 *n*-3) and docosahexaenoic acid (DHA, 22:6 *n*-3) inhibit interleukin-6 (IL-6) and interleukin-1β (IL-1β) production in human macrophages [17,18]. It has been suggested that *n*-3 LC-PUFA may play a role as nutritional therapeutic ingredients for the prevention and treatment of inflammatory diseases [19]. Certain polyphenols, pigments, and other plant bioactives have also demonstrated anti-inflammatory activity both *in vitro* and *in vivo* [20,21,22] suggesting potential for dietary ingredients to ameliorate symptoms of chronic inflammatory diseases.

Marine and freshwater algae produce a number of bioactive compounds including peptides, carbohydrates, phenols, pigments, and lipids with potential health benefits [23,24]. For example, marine macroalgae (seaweeds) typically contain around 2%–5% lipid on a dry weight basis, where up to 70% of the total fatty acids can be PUFA [25,26]. Some microalgae are reported to contain high protein, lipid (up to 60% of dry weight) and PUFA contents (up to 60% of total fatty acids), and have been exploited for their protein and lipid content for a number of nutritional and industrial purposes [27,28]. Both micro- and macroalgae may thus act as sources of essential vitamins, minerals, and bioactive pigments.

Algae potentially represent an abundant and underexplored resource of health promoting functional food ingredients, which may be particularly useful in the Western diet, which is otherwise rich in meat products. Additionally, fish stocks are facing global decline alongside increasing toxin accumulation due to pollution [29,30,31], hence algae may constitute a more economical and sustainable resource for dietary *n*-3 LC-PUFA and other bioactive marine ingredients [32]. Indeed, a number of whole algae-extracts and algae-derived compounds have exhibited anti-inflammatory activity through the inhibition of pro-inflammatory cytokine and eicosanoid production, and reduction of expression of pro-inflammatory genes [33,34,35,36,37,38]. Yang *et al.* found that extracts from five Korean seaweeds (*Laurencia okamurae*, *Grateloupia elliptica*, *Sargassum thunbergii*, *Gloiopeltis furcata*, and *Hizikia fusiformis*) reduced inflammation in the mouse macrophage cell line RAW 264.7 through inhibition of the pro-inflammatory eicosanoid prostaglandin E2 (PGE2) and the pro-inflammatory cytokines IL-6 and tumor-necrosis factor α (TNFα) [39]. Furthermore, Nauroth *et al.* demonstrated the anti-inflammatory activity of microalgal oil when fed to rats [40]. In addition, Banskota *et al.* reported anti-inflammatory effects of lipid extracts of *Tetraselmis chuii*, *Chlorella sorokiniana* and *Chondrus crispus* in RAW 264.7 macrophages [41,42,43]. Accordingly, there is evidence to suggest that algal extracts may be beneficial as functional food ingredients to control inflammation.

The aim of this study was to evaluate the anti-inflammatory activity of three algal species from the Irish coast and a microalga and to identify their potential as anti-inflammatory functional food ingredients. To this end, lipid extracts of the edible and commercially valuable red macroalgae *Porphyra dioica*, *Palmaria palmata*, *Chondrus crispus* (all Rhodophyta) and *Pavlova lutheri* (Haptophyta) were characterized for their fatty acid, pigment, lipid and LC-PUFA partitioning profiles. Subsequently the anti-inflammatory bioactivities of these extracts were assessed through their potential to influence cytokine production and inflammatory gene expression in human THP-1 macrophages.

## 2. Results

### 2.1. Fatty Acid Composition of Algal Lipid Extracts

The fatty acid composition, expressed as % of total fatty acids of the algal lipid extracts are shown in Table 1. Each extract contained a broad spectrum of medium to long-chain saturated and unsaturated fatty acids. Total saturated fatty acid (SFA) content was similar across all four algal species, ranging from 28% to 32.4% of total saturated fatty acids. SFA content was dominated by palmitic acid (16:0) in the three seaweed species (*P. palmata*, *P. dioica* and *C. crispus*) ranging from 22.3% to 28.1%, whereas the dominant SFA in the *P. lutheri* (microalgal) extract was myristic acid (14:0) at 14.3%, followed by 12.8% of 16:0.

**Table 1:** Fatty acid composition of the four algal lipid extracts.

Extracts (% Total Fatty Acids)	*Pavlova lutheri*		*Palmaria palmata*		*Porphyra dioica*		*Chondrus crispus*	
	Average	SD	Average	SD	Average	SD	Average	SD
*Saturated fatty acids (SFA)*								
12:0	0.19	0.14	0.16	0.05	0.32	0.13	0.42	0.15
14:0	14.26	0.47	4.51	0.35	0.53	0.10	1.98	0.39
16:0	12.84	0.19	22.27	0.40	28.12	1.19	27.33	0.50
18:0	0.68	0.02	1.69	0.07	2.16	0.11	2.71	0.05
Sum of SFAs	27.97	0.61	28.63	0.63	31.14	1.51	32.44	0.90
*Monounsaturated fatty acids (MUFA)*								
14:1	0.55	0.01	0.51	0.07	0.54	0.12	0.48	0.11
15:1	1.66	0.09	1.53	0.14	1.51	0.02	1.69	0.27
16:1 *n*-7	14.36	0.45	1.17	0.20	2.37	0.09	0.58	0.08
18:1 *n*-9	2.60	0.07	2.18	0.02	1.72	0.02	5.47	0.02
18:1 *n*-7	0.31	0.05	0.73	0.01	0.68	0.00	0.59	0.01
20:1 *n*-9	n.d.		n.d.		1.62	0.04	n.d.	
Sum of MUFAs	19.49	0.54	6.12	0.11	8.44	0.06	8.82	0.24
*Polyunsaturated fatty acids (PUFA)*								
16:2 *n*-6	0.85	0.22	n.d.		n.d.		n.d.	
16:2 *n*-4	1.68	0.11	n.d.		n.d.		n.d.	
16:4 *n*-3	0.62	0.13	n.d.		n.d.		n.d.	
18:2 *n*-6	0.59	0.02	0.65	0.01	1.64	0.01	1.58	0.05
18:3 *n*-6	2.09	0.04	0.26	0.01	0.56	0.01	0.49	0.01
18:3 *n*-3	1.13	0.01	0.65	0.01	1.34	0.02	0.23	0.00
18:4 *n*-3	4.98	0.08	2.16	0.04	1.58	0.01	0.25	0.01
20:2 *n*-6	n.d.		1.06	0.15	0.87	0.02	0.37	0.05
20:3 *n*-6	n.d.		n.d.		2.21	0.35	0.19	0.16
20:4 *n*-6	0.60	0.02	0.67	0.01	3.03	0.07	19.85	0.82
20:5 *n*-3	27.67	0.37	57.94	0.89	46.35	0.91	33.47	0.21
22:5 *n*-3	1.17	0.20	n.d.		n.d.		n.d.	
22:6 *n*-3	10.47	0.38	1.15	0.24	n.d.		n.d.	
Sum of PUFAs	51.85	0.84	64.55	0.87	57.57	1.35	56.42	1.29
*n*-3	46.04	1.05	61.91	0.71	49.26	0.93	33.94	0.20
*n*-6	4.13	0.18	2.64	0.17	8.31	0.44	22.47	1.10
Ratio *n*-6/ *n*-3	0.09	0.01	0.04	0.00	0.17	0.01	0.66	0.03
Others	0.69	0.30	0.70	0.12	2.85	0.14	2.32	0.61

Data are expressed as % of total fatty acid methyl esters (FAME) and are presented as mean values ± standard deviation (SD); n.d., not detected.

Total monounsaturated fatty acid (MUFA) content ranged from 6.1% to 8.8% in the three red seaweed species whereas *P. lutheri* displayed 19.5% of total fatty acids as MUFA, of which palmitoleic acid (16:1 *n*-7) was most abundant with 14.4%. Oleic acid (18:1 *n*-9) was another MUFA present at high levels, ranging from 1.7% in *P. dioica* to 5.5% in *C. crispus*.

Polyunsaturated fatty acids (PUFA) were more dominant than SFA and MUFA in all extracts, ranging from 51.9% to 64.6% of total fatty acids. Of these PUFA, *n*-3 fatty acids were present in greater quantities than *n*-6 PUFA in all extracts. EPA was the most abundant PUFA and the most abundant fatty acid in all extracts, ranging from 27.7% in *P. lutheri* to 57.9% in *P. palmata.* DHA accounted for 10.5% of total fatty acids in *P. lutheri* and was present in undetectable or very low quantities in all other extracts. Of note, *P. lutheri* contained 5% stearidonic acid (SDA, 18:4 *n*-3). Additionally the *n*-6 LC-PUFA arachidonic acid (ARA, 20:4 *n*-6) was present in very high quantities in *C. crispus* at 19.9% of total fatty acids. The ratio of *n*-6/*n*-3 fatty acids was <0.2 in *P. lutheri*, *P. palmata* and *P. dioica* (0.09, 0.04, and 0.17, respectively) whereas *C. crispus* had an *n*-6/*n*-3 ratio of 0.66 due to its higher proportion of *n*-6 ARA.

### 2.2. Pigments

Pigment contents of the different extracts are detailed in Table 2. Individual pigment values are expressed as % molar and total pigment values are expressed as % of dry weight crude extract. Pigments concentrations were relatively low but substantial in all extracts, ranging from 4.9% to 6.8% of the crude extract. Chlorophyll *a* and its degradation products were most abundant in all extracts accounting for 41.1%–53% of total pigments. β-Carotene and zeaxanthin were the only other pigments detected in the red seaweed extracts ranging from 19.2% to 30.2% and from 22.8% to 27.5%, respectively. Fucoxanthin and its degradation products, as well as diadinoxanthin and diatoxanthin and their degradation products, were also present in relatively high proportions (26.9% and 16.1%, respectively) in *P. lutheri*, alongside a number of other pigments (*i.e.*, β-carotene, *cys*-fucoxanthin, and chlorophyll *c*).

**Table 2:** Pigment composition of four algal lipid extracts.

Extracts	*Pavlova lutheri*		*Palmaria palmata*		*Porphyra dioica*		*Chondrus crispus*	
Pigments (% Molar)	Average	SD	Average	SD	Average	SD	Average	SD
chlorophyll *a* + deg.	41.10	0.05	52.03	0.05	53.02	0.37	43.95	0.30
β-carotene	7.05	0.09	25.17	0.34	19.15	0.27	30.22	0.09
zeaxanthin	n.d.		22.80	0.38	27.53	0.59	25.84	0.40
diadinoxanthin + diatoxanthin + deg.	16.11	0.03	n.d.		n.d.		n.d.	
*cys*-fucoxanthin	5.51	0.01	n.d.		n.d.		n.d.	
chlorophyll *c* + deg.	3.36	0.08	n.d.		n.d.		n.d.	
fucoxanthin + deg.	26.87	0.07	n.d.		n.d.		n.d.	
Pigments (% crude extract)	6.80	0.05	6.03	0.41	5.13	0.06	4.87	0.04

Data are expressed as % molar and are presented as mean values ± standard deviation (SD); Total pigments are expressed as % of dry weight of crude extract; n.d., not detected; deg., degradation products.

### 2.3. Lipid Class Composition and LC-PUFA Partitioning

**Figure 1:**
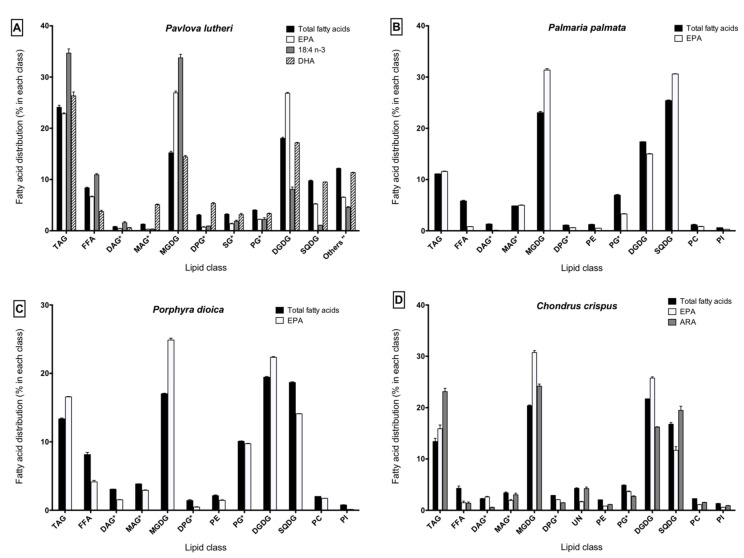
Distribution of fatty acids within lipid classes in (**A**) *Pavlova lutheri*; (**B**) *Palmaria palmata*; (**C**) *Porphyra dioica*; (**D**) *Chondrus crispus*. * Identified using *R*_f_ values from the literature using similar migration solvent systems without using standards; " Sum of other unidentified polar lipids, *i.e.*, phospholipids and betaine lipids; No symbol: Identified using *R*_f_ values from the literature and standards; EPA: eicosapentaenoic acid; DHA: docosahexaenoic acid; TAG: triacylglycerol; FFA: free fatty acids; DAG: diacylglycerols; MAG: monoacylglycerols; MGDG: monogalactosyldiacylglycerols; DPG: diphosphatidylacylglycerols; SG: acylated sterol glycosides; PE: phosphatidylethanolamines; PG: phosphatidylglycerols; DGDG: digalactosyldiacylglycerols; SQDG: sulfoquinovosyldiacylglycerols; PC: phosphatidylcholines; PI: phosphatidylinositols; UN: unidentified.

The lipid class composition of each lipid extract and the distribution of the major LC-PUFA (SDA, ARA, EPA and DHA) within the different lipid fractions are detailed in Figure 1.

In *P. lutheri*, the triacylglycerol (TAG) and glycolipid families represent together 67.1% of total fatty acids. The neutral lipid fraction, composed by TAG (24.1%), diacylglycerols (DAG, 0.8%), monoacylglycerols (MAG, 1.3%), and free fatty acids (FFA, 8.4%) accounted for 34.5% of total fatty acids. The polar lipid fraction was mainly composed of monogalactosyldiacylglycerols (MGDG, 15.2%), digalactosyldiacylglycerols (DGDG, 18.0%) and sulfoquinovosyldiacylglycerols (SQDG, 9.8%), which represent a total glycolipid proportion of 43% of total fatty acids. Other unidentified lipid classes accounting together for 12.2% of total fatty acids were also found in the polar lipid fraction.

In the red seaweeds (*P. palmata*, *P. dioica*, *C. crispus*), TAG and FFA represent the main neutral lipid classes with 11.1%–13.4% and 4.3%–8.1% of total fatty acids, respectively. The polar fraction was essentially composed of MGDG (17.0%–23.1%), DGDG (17.4%–21.7%) and SQDG (16.8%–25.4%), representing 71.6%–76.9% of total fatty acids. Phospholipids (11.1%–16.4%) such as diphosphatidylacylglycerols (DPG), phosphatidylethanolamines (PE), phosphatidylglycerols (PG), phosphatidylcholines (PC) and phosphatidylinositols (PI) were also found in the polar fraction of the red macroalgae.

Despite small differences between species, LC-PUFA were mainly partitioned into the glycolipid (MGDG, DGDG and SQDG) and TAG fractions. In *P. lutheri*, it might be important to note that DHA was also substantially incorporated into the other unidentified lipid classes (11.3% of the total DHA); EPA was also incorporated into the PG of *P. dioica* (10.1% of the total EPA).

### 2.4. Inhibition of Inflammatory Cytokine Production in Lipopolysaccharide (LPS)-Stimulated THP-1 Macrophages

**Figure 2:**
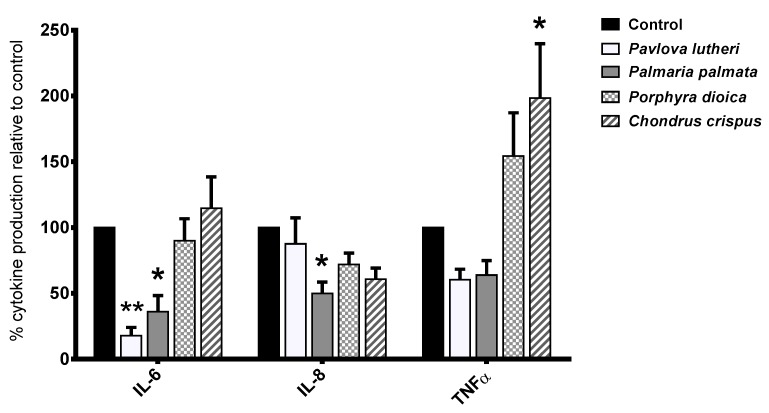
Effects of algal lipid extracts exposure on interleukin-6 (IL-6), IL-8 production and tumor necrosis factor α (TNFα) production in lipopolysaccharide (LPS)-stimulated THP-1 macrophages. THP-1 macrophages were exposed to the respective algae-lipid extracts or the vehicle control (dimethyl sulfoxide, DMSO) for 24 h and incubated with 100 ng·mL^−1^ LPS for a further 24 h. Values represent mean ± standard error (SE) normalized to DMSO control. * *p* ≤ 0.05; ** *p* ≤ 0.01.

The effects of THP-1 macrophage exposure to the four algal lipid extracts on lipopolysaccharide (LPS)-stimulated IL-6, IL-8 and TNFα production are shown in Figure 2. Following 24 h-exposure to *P. lutheri* lipid extract, the production of the pro-inflammatory cytokine IL-6 (*p* < 0.01) was significantly downregulated in comparison to the carrier control. Incubation of the THP-1 cells with *P. palmata* lipid extract significantly reduced production of IL-6 (*p* < 0.05) and IL-8 (*p* < 0.05), whereas incubation with *C. crispus* lipid extract led to significantly increased production of TNFα relative to the carrier control (*p* < 0.05). *P. dioica* did not appear to significantly influence the production of any of the examined cytokines. There were no significant responses in IFNγ IL-12p70, IL-10 or IL-1β production by THP-1 cells following exposure to any of the algal lipid extracts.

**Table 3:** Effect of algal lipid extracts treatment followed by lipopolysaccharide (LPS) exposure on the differential expression of a panel of inflammatory genes in THP-1 macrophages.

Extract	Gene Symbol	Name	Fold Change	Gene Description and Function
*P. lutheri*	*TLR8*	Toll-like receptor 8	−3.33	PAMP recognition and activation of innate immunity. Mediates cytokine production through activation of NF-κB
	*TLR1*	Toll-like receptor 1	−4.16	Interacts with TLR2 for immune activation through PAMP recognition.
	*TRAF5*	TNF receptor-associated factor 5	−2.69	Mediates signal transduction of the TNF receptor family. Mediates NF-κB and JNK activation
	*MAP3K1*	Mitogen-activated protein kinase 1	−2.63	Activates protein kinase signal transduction cascade such as the ERK and JNK kinase pathways and NF-κB pathway.
	*PTGER1*	Prostaglandin E receptor 1	+2.58	Encodes a receptor for PGE2. Down-regulates COX2 and hence resolves inflammation.
*P. palmata*	*TLR8*	Toll-like receptor 8	−2.91	PAMP recognition and activation of innate immunity. Mediates cytokine production through activation of NF-κB
	*TLR1*	Toll-like receptor 1	−3.99	Interacts with TLR2 for immune activation through PAMP recognition.
	*TRAF5*	TNF receptor-associated factor 5	−2.73	Mediates signal transduction of the TNF receptor family. Mediates NF-κB and JNK activation
	*NOS2*	Nitric oxide synthase 2	+2.83	Encodes nitric oxide synthase which mediates tumoricidal and bactericidal actions in macrophages
*P. dioica*	*CCR1*	Chemokine (C-C motif) receptor 1	−3.46	Acts as a receptor for chemokines such as MIP-1α and MCP-3 which assist in immune cell recruitment
	*TLR8*	Toll-like receptor 8	−3.29	PAMP recognition and activation of innate immunity. Mediates cytokine production through activation of NF-κB
	*TLR2*	Toll-like receptor 2	−4.25	Interacts with TLR1 for PAMP recognition leading to NF-κB activation and cytokine production
	*TLR1*	Toll-like receptor 1	−2.5	Interacts with TLR2 for immune activation through PAMP recognition.
	*TRAF5*	TNF receptor-associated factor 5	−3.37	Mediates signal transduction of the TNF receptor family. Mediates NF-κB and JNK activation
	*TNFSF18*	TNF (ligand) superfamily, member 18	−2.93	Regulates T-cell activities by lowering the threshold for T-cell activation.
	*TRAF6*	TNF receptor-associated factor 6	−2.99	Mediates signal transduction from TNF receptors and Toll/IL-1 receptors.
	*MAP3K1*	Mitogen-activated protein 3 kinase 1	−3.63	Activates protein kinase signal transduction cascade such as the ERK and JNK kinase pathways and NF-κB pathway.
	*STAT3*	Signal transducer and activator of transcription 3	−3.56	Activated by cytokines to create transcription factors that form part of JAK-STAT signaling cascade.
	*CCR5*	Chemokine (C-C motif) receptor 5	−2.72	Chemokine receptor, expressed in macrophages involved in immune cell recruitment.
	*TLR4*	Toll-like receptor 4	−4.44	PAMP recognition and activation of inflammatory cascade. Specifically recognises LPS
	*IL6R*	Interleukin 6 receptor	−2.54	Binds with low affinity to the inflammatory cytokine IL-6 regulating immune response and acute phase reactions.
	*PTGER1*	Prostaglandin E receptor 1	+2.7	Encodes a receptor for PGE2. Down-regulates COX2 and hence resolves inflammation.
*C. crispus*	*IL23*	Interleukin 23	−4.19	Activates STAT4 and stimulates production of IFNγ Associated with autoimmune inflammation and tumorigenesis.
	*TLR8*	Toll-like receptor 8	−2.59	PAMP recognition and activation of innate immunity. Mediates cytokine production through activation of NF-κB
	*CCL17*	Chemokine (C-C motif) ligand 17	−3.33	Encodes a cytokine that is a chemotactic factor for T-lymphocytes. Recruitment and activation of mature T-cells
	*TLR1*	Toll-like receptor 1	−3.25	Interacts with TLR2 for immune activation through PAMP recognition.
	*TRAF5*	TNF receptor-associated factor 5	−2.76	Mediates signal transduction of the TNF receptor family. Mediates NF-κB and JNK activation
	*TRAF6*	TNF receptor-associated factor 6	−3.04	Mediates signal transduction from TNF receptors and Toll/IL-1 receptors.

Values are expressed as fold change in gene expression relative to the dimethyl sulfoxide (DMSO) vehicle control. Negative values represent gene down-regulation and positive values represent gene up-regulation. A fold difference cut-off point was set at ≥2.5. PAMP: pathogen-associated molecular pattern; NF-κB: nuclear factor kappa-light-chain-enhancer of activated B cells; TNF: tumor necrosis factor; JNK: c-Jun *N*-terminal kinase; ERK: extracellular signal-regulated kinase; PGE2: prostaglandin E2; COX2: cyclooxygenase 2; STAT4: signal transducer and activator of transcription 4; MIP-1α: macrophage inflammatory proteins-1α; MCP-3: monocyte-specific chemokine-3; JAK: Janus kinase; IFNγ: interferon gamma.

### 2.5. Inhibition of Inflammatory Gene Expression by Algal Lipid Extracts

In order to assess the anti-inflammatory effects of the algal lipid extracts in the THP-1 macrophages, quantitative expression of a panel of 92 inflammatory marker genes was evaluated. Of the 92 target genes analyzed, 16 genes were differentially expressed following exposure to the algal lipid extracts as shown in Table 3. mRNA abundances of toll-like receptor 1 (*TLR1*) and toll-like receptor 8 (*TLR8*) and TNF receptor associated factor 5 (*TRAF5*) were consistently downregulated by the lipid extracts from all four algal species. In addition, *P. dioica* extract downregulated nine other pro-inflammatory genes including pro-inflammatory markers toll-like receptor (*TLR4*) and signal transducer and activator of transcription 3 (*STAT3*). Other genes that were downregulated by algal lipid extracts predominantly represent pro-inflammatory pathways such as those sharing toll-like receptors, TNF receptor associated factors, chemokine, cytokines and their receptors. While the lipid extracts from all four algal species predominantly indicated an inhibitory effect on the pro-inflammatory genes, the up-regulation of *NOS2* by *P. palmata* and *PTGER1* by *P. lutheri* and *P. dioica* indicated activation of a pro-inflammatory response which may be due to the fact that the crude lipid extracts may contain certain compounds with pro-inflammatory activities.

### 2.6. Validation of PCR Array Results through Quantitative PCR (qPCR)

**Figure 3:**
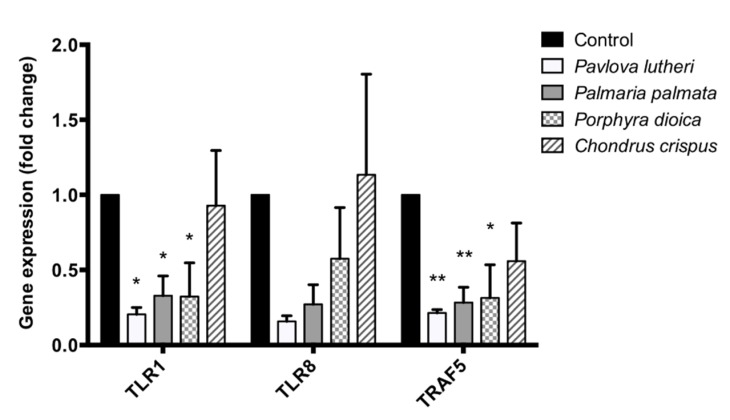
Validation of PCR array results through quantitative PCR. Effects of algal lipid extracts exposure on expression of *TLR1*, *TLR8*, and *TRAF5* genes in lipopolysaccharide (LPS)-stimulated THP-1 macrophages. * *p* ≤ 0.05. ** *p* ≤ 0.01.

For the validation purpose, three genes (*TLR1*, *TLR8* and *TRAF5*) were selected for qPCR analysis given that these genes were consistently downregulated by all four algal lipid extracts as evident in the PCR array data. The oligonucleotide sequences for the primers used for quantitative PCR for these genes are outlined in Table 4. The effects of the four algal lipid extracts on *TLR1*, *TLR8* and *TRAF5* gene expression in LPS-stimulated macrophages are shown in Figure 3. Compared to the carrier control, exposure of the THP-1 cells to the lipid extracts from *P. lutheri*, *P. palmata* and *P. dioica* resulted in inhibition of *TLR1*, *TLR8*, and *TRAF5* mRNA abundances indicating consistency of the PCR array data which were generated through gene expression analysis in the pooled cDNA samples. For the *C. crispus* lipid extract, a reduction in the expression of these genes was least pronounced due to a high standard deviation (SD) because of one individual replicate.

**Table 4:** Oligonucleotide sequences of forward and reverse primers for quantitative real-time PCR (qPCR).

Gene Name	Primers	Nucleotide Sequence (5′ to 3′)	*T*_m_ (°C)
*GAPDH*	Forward	ACAGTTGCCATGTAGACC	55.7
	Reverse	TTTTTGGTTGAGCACAGG	59.9
*TLR1*	Forward	CCCTACAAAAGGAATCTGTATC	58.2
	Reverse	TGCTAGTCATTTTGGAACAC	57.8
*TLR8*	Forward	TGGAAAACATGTTCCTTCAG	60.1
	Reverse	TGCTTTTTCTCATCACAAGG	60.4
*TRAF5*	Forward	GGAATGGCTTATTCAGAAGAG	59.4
	Reverse	CCACAAACTGGTACTCTATAC	52.8

*T*_m_: Melting temperature.

## 3. Discussion

In this study we investigated the anti-inflammatory activity of lipid extracts of four algal species in human THP-1 macrophages. The fatty acid, pigment and lipid class, and LC-PUFA partitioning profiles of three red seaweeds (*Palmaria palmata*, *Porphyra dioica*, *Chondrus crispus*) and one microalga (*Pavlova lutheri*) were identified, which contained high proportions of *n*-3 PUFA (34%–42% of total fatty acids) and 5%–7% pigments including chlorophyll *a*, β-carotene, and fucoxanthin. When exposed to human THP-1 macrophages for 24 h, lipid extracts of *P. lutheri* (*p* < 0.01) and *P. palmata* significantly (*p* < 0.05) suppressed LPS-induced production of the pro-inflammatory cytokine IL-6, compared with the untreated control. Similarly, *P. palmata* (*p* < 0.05) lipid extract significantly reduced production of the pro-inflammatory cytokine IL-8 compared with the control. All extracts inhibited the expression of a number of inflammatory genes in THP-1 macrophages, including those involved in toll-like receptor activity and chemokine and cytokine-linked signaling pathways. These results suggest that the algal lipid extracts examined have potential to suppress inflammation through inhibition of inflammatory cytokine production and down-regulation of genes involved in a number of inflammatory signaling pathways.

The results provide additional compositional data of lipid extracts from these four algal species based on fatty acid, pigment, and lipid profiles. In accordance with previous reports [18,39], this study demonstrated algal LC-PUFA are mainly partitioned into complex polar lipids constituting the membranes and in a less extent into TAG of some species with the ability to accumulate TAG containing LC-PUFA. Each algal species contained high proportions of *n*-3 PUFA (34%–62% total fatty acids), with EPA being the most abundant fatty acid in all extracts, ranging from 28% to 58% of total fatty acids. Indeed, *P. lutheri* has been widely examined for the ability to produce high concentrations of both EPA and DHA, and optimized culture conditions have been studied to maximize *n*-3 LC-PUFA production [44,45,46,47]. Similarly, red seaweeds, including those examined in this study, have also been identified as rich sources of EPA and desirable *n*-6:*n*-3 PUFA ratios [26]. Macroalgal lipid composition, however, is susceptible to seasonal variation and it has been shown that pigment and LC-PUFA levels are optimized under low light and temperature conditions as observed, for example, in winter [26].

Indeed, *n*-3 LC-PUFA within algae are primarily incorporated into complex lipid classes such as MGDG, DGDG, and SQDG [24,48,49]. Banskota *et al.* have previously demonstrated that polar lipids isolated from red algae demonstrate stronger anti-inflammatory activity than pure EPA isolated from the same species [43], suggesting that the entire polar lipid structure contributes to anti-inflammatory activity. Hence, the polar lipid structures, into which the fatty acids are incorporated in the species examined in this study, may contribute to the observed inhibition of cytokine production and inflammatory gene expression. Interestingly however, the *C. crispus* extract significantly increased production of TNFα, a proinflammatory cytokine. The reason for this may be due to the synergistic effect of a number of bioactive pigments and fatty acids in the extract including ARA, which was present in high quantities in the *C. crispus* extract and which is recognized as a pro-inflammatory fatty acid [50]. Chlorophyll *a*, β-carotene, fucoxanthin, and zeaxanthin have all previously demonstrated anti-inflammatory activity [22,51,52,53] and hence may have contributed to the anti-inflammatory activity observed in this study. Chlorophyll *a* is of particular interest as it was present from 2.1 to 3.1 g/100 g crude extract. However, due to the extremely diverse fatty acid and pigment composition of the extracts, it is not possible to attribute the observed biological effects to any individual fatty acids or pigments. It is likely that there are synergistic effects between many bioactives including fatty acids and pigments which contribute to the observed anti-inflammatory effects in a number of pathways.

The results presented here expand on previous findings showing that algal extracts inhibit inflammation *in vitro*. Yang *et al.* reported anti-inflammatory activity in five Korean seaweed extracts in mouse RAW 264.7 macrophages, in which IL-6, TNFα and prostaglandin E2 (PGE2) production were inhibited [39]. Hwang *et al.* reported similar results for an extract from *Sargassum hemiphyllum* in the same cell line [33]. In addition, both Khan *et al.* and Ishihara *et al.* isolated pure PUFA from *Undaria pinnatifida* extracts and also demonstrated their anti-inflammatory activity [35,54]. Our data provide novel evidence for the anti-inflammatory activity of algal extracts and show for the first time that *P. lutheri*, *P. palmata*, *P. dioica* and *C. crispus* lipids can inhibit induced inflammatory response in human macrophages. Hence these results suggest that these algal species, as dietary ingredients, may have the potential to suppress chronic inflammation *in vivo*; however further work is warranted to assess if the *in vitro* activity is translated to an *in vivo* model.

It is likely that the crude mix of fatty acids, polar lipids and pigments in the extracts influenced a number of inflammatory pathways in the cell, as seen by the wide range of genes altered in the gene expression array. LPS is an agonist of toll-like receptor 4 (TLR4), a membrane-bound receptor that activates the nuclear factor kappa-light-chain-enhancer of activated B cells (NF-κB) pathway that leads to a pro-inflammatory response [55]. In the present study, treatment of the macrophages with *P. dioica* extract downregulated expression of *TLR4* by more than four folds, indicating inhibition of NF-κB mediated pro-inflammatory pathway, however *TLR4* expression was not altered by any of the other extracts. In the present study, a number of genes involved in NF-κB signaling were significantly downregulated by the algal lipid extracts. Expression of *TLR1*, a toll-like receptor involved in pathogen-associated molecular pattern (PAMP) recognition and NF-κB activation, was downregulated by all extracts and has previously been shown to be altered by unsaturated and saturated fatty acids [56]. STAT3 and MAP3K1 are signaling molecules also involved in NF-κB activation through the JAK-STAT (Janus kinase-signal transducer and activator of transcription) and JNK kinase pathways respectively, which have been shown to be susceptible to change in expression by PUFA and a carotenoid extract of *Dunaliella salina* [57,58,59]. These genes were significantly downregulated by lipid extracts of *P. dioica* and *P. lutheri*, further elucidating a role that such extracts play in the downregulation of the NF-κB pathway. Moreover, a lipid extract from the cyanophyte *Nostoc commun*e which contained *n*-3 PUFA, β-carotene and a number of other bioactives has previously been demonstrated to inhibit inflammatory response in macrophages through inhibition of NF-κB signaling.

In this study, we also evaluated the effect of algae-derived lipid extract on the expression of *NOS2* gene in LPS-stimulated THP-1 cells. While the mRNA levels of *NOS2* gene were not affected by the extracts from *P. lutheri*, *P. dioica* and *C. crispus*, the expression was increased by the *P. palmata* lipid extracts. A higher *NOS* gene expression is likely to increase the level of inducible nitric oxide (iNOS) that mediates pro-inflammatory response. LPS was reported to stimulate iNOS production in THP-1 cells [60]. In the present experiment, the high *NOS2* gene expression by *P. palmata* lipid extracts could be due to the failure of this extract to suppress the LPS induced *NOS2* gene expression and/or exaggerations of the iNOS mediated pro-inflammatory response in THP-1 cells. Due to the presence of crude and complex mixture of fatty acids and pigments in the extract, it was difficult to speculate the precise bioactive compound/s that drives such an increase in the *NOS2* gene expression or whether such a response is translated into higher iNOS leading to a pro-inflammatory response. It is likely that certain bioactive pigments such as β-carotene, and fatty acids, such as arachidonic acid, which have previously demonstrated immune enhancing activities [61,62], and that are present in the crude lipid extracts of *P. palmata* contributed to an increase in *NOS2* expression, as was previously reported for an extract of *Ulva rigida* in murine RAW 264.7 macrophages [63], Hence, isolation and purification of individual bioactive compounds from the algal crude lipid extract and understanding their molecular mode of action will provide further details of the immune modulatory potential of these novel bioactives.

The present study explored the anti-inflammatory bioactivity of algal lipid extracts containing a crude mixture of fatty acids, pigments, and polar lipids, each of which may affect a number of related and unrelated gene expression and cytokine production, and the identification of individual anti-inflammatory pathways affected by the bioactive component; their precise molecular mode of action warrants further study.

Of the species studied, lipid extract from *C. crispus* appeared to display the lowest anti-inflammatory potential. This may be due to its high content of *n*-6 ARA, which has been associated with pro-inflammatory properties. Extracts from *P. lutheri* and *P. palmata* were the most potent extracts in inhibiting inflammatory cytokine production, whereas those from *P. dioica* inhibited the expression of more inflammatory genes than any of the other species tested. As microalgae tend to have much higher lipid contents than macroalgae, are more efficient producers of *n*-3 LC-PUFA and contain more diverse pigment profiles, as observed here, *P. lutheri* may pose the best potential as an anti-inflammatory functional food ingredient.

In conclusion, we demonstrated for the first time that lipid extracts from *P. lutheri*, *P. palmata*, *P. dioica* and *C. crispus* downregulated LPS-induced pro-inflammatory responses in human macrophages through inhibition of IL-6 and IL-8 production. This anti-inflammatory mechanism of algal lipid extract is most likely due to alteration in the expression of genes that encode signaling molecules linked to the NF-κB pathway. We hypothesize that the potent anti-inflammatory effect shown by these extracts is attributable to a synergistic effect of bioactive compounds such as pigments and *n*-3 LC-PUFA incorporated into complex lipid structures which have previously been shown to exert similar anti-inflammatory effects. However further work is warranted to identify which compound exert the strongest biological effects. The bioactivity of these novel algal-lipid extracts as anti-inflammatory functional food ingredients in chronic inflammation-linked metabolic diseases should be further explored.

## 4. Experimental Section

### 4.1. Materials

All chemicals and reagents were purchased from Sigma-Aldrich Ireland Ltd. (Arklow, Republic of Ireland) unless otherwise stated. Tissue culture plastics were purchased from Sarstedt (Wexford, Republic of Ireland). The human THP-1 monocytic cell line was purchased from the American Tissue Culture Collection (ATCC, LGC Standards, Middlesex, UK). Working solutions of bacterial lipopolysaccharide (LPS; *Escherichia coli* 055:B5) and phorbol-12-myristate-13-acetate (PMA) were stocked in RPMI 1640 medium.

### 4.2. Collection of Macroalgae and Biomass Preparation

Samples of the three red algae *Chondrus crispus*, Stackhouse (Gigartinales,), *Palmaria palmata* (L.) F. Weber & D. Mohr (Palmariales) and *Porphyra dioica*, J. Brodie & L. M. Irvine (Bangiales) were collected in Galway Bay (Spiddal 53°14′22″ N, 9°18′52″ W) during March 2013. Algae were placed on ice for approximately 2–3 h during transportation to the laboratory, rinsed with fresh water and cleaned of all visible epiphytes, if present, and frozen at −20 °C. Algal material was lyophilised using Labconco Freezone^®^ (Kansas City, MO, USA) 6 freeze-dryer system and ground to fine powder for lipid extraction.

Algal species were identified and extracts supplied by the Algal Biosciences Research Group of Dagmar Stengel (Botany and Plant Science, School of Natural Sciences, Ryan Institute for Environment, Marine and Energy, National University of Ireland Galway).

### 4.3. Microalgal Strain, Culture Conditions and Biomass Preparation

An axenic strain of *Pavlova lutheri* CCAP 931/6, formerly renamed *Diacronema lutheri* [64], was obtained from the Culture Collection of Algae and Protozoa at the Scottish Marine Institute (SAMS Research Services Ltd., Oban, Scotland, UK) and cultivated in the laboratory at the National University of Ireland Galway.

*P. lutheri* were cultivated under batch conditions on F/2-RSE medium in three 15 L polycarbonate Nalgene^®^ culture vessels at 15 °C and under continuous illumination (100 μmol photons m^−2^·s^−1^), provided by lumilux cool daylight fluorescent lamps (OSRAM L18W/865, Munich, Germany). F/2-RSE medium, a modified version of Guillard’s (1975) F/2 medium [65] where filtered seawater is substituted by ReefSalt (H2Ocean, Pro+, Essex, IG6 3UT, England, UK), was used and complemented, as described by Guihéneuf *et al.* [66], with few modifications, a higher initial NaNO_3_ concentration (350 mg·L^−1^) and NaHCO_3_ supplementation (1 g·L^−1^). Cultures were agitated by air-bubbling (~0.03%–0.04% CO_2_).

*P. lutheri* cells were harvested after 50 days (late-exponential growth phase), just after nitrate-depletion was reached on day 40 in order to allow maximum accumulation of oil containing *n*-3 LC-PUFA as described by Guihéneuf *et al.* [66], by centrifuging using a modified Alistar milk cream separator (80 L·h^−1^). Prior to lipid extraction, the fresh algal paste collected was frozen at −20 °C and freeze-dried as previously described.

### 4.4. Lipid Extraction

Total lipids were extracted by solid-liquid procedure using a modified version of Bligh and Dyer’s method [67]. Briefly, freeze-dried ground macroalgal biomass or freeze-dried microalgal powder was extracted with methanol:chloroform 1:1 (*v*/*v*) in a glass bottle closed under nitrogen gas (N_2_) to limit oxidation, and stirred overnight at 4 °C. The first extract obtained was filtered and the remaining biomass re-extracted 2–3 times similarly. All the filtered-extracts were then combined and Milli-Q water added to give a final chloroform:methanol:water ratio of 1:1:0.9 (*v*/*v*/*v*). After phase separation, the organic phase containing lipids (lower phase) was collected, and the extraction was repeated 2–3 times by adding chloroform to the remaining methanol:water phase. The combined organic phases containing the total crude lipid extract were then evaporated to dryness using a rotavapor (BÜCHI R-210 equipped with a Heating Bath B-491 and a Vacuum Pump V700, BÜCHI Labortechnik AG, 9230 Flawil, Switzerland) under reduced pressure at 40 °C.

### 4.5. Lipid Class Separation

The lipid class separation procedure combined the method described by Sukenik *et al.* [68] using Sep-Pak silica cartridges to separate the neutral and polar lipids, and thin-layer chromatography procedures adapted from Williams [69], Christie [70], and Henderson and Tocher [71] to separate individual lipid classes.

#### 4.5.1. Lipid Separation by Silica Cartridge Chromatography

A known amount of total crude lipid extract dissolved in chloroform was fractionated on reversed phase silica gel columns (Bond Elut™ JR-SI 500 mg, Agilent Technologies, Santa Clara, CA, USA) after an activation step with 5 mL of methanol followed by 5 mL of chloroform. Neutral lipids were eluted using 20 mL of chloroform, while polar lipids were eluted with 20 mL of methanol [68]. The neutral and polar fractions obtained were evaporated to dryness using a rotary evaporator under reduced pressure at 40 °C and dissolved in a small volume of chloroform or methanol, respectively.

#### 4.5.2. Neutral and Polar Lipid Fractionation by Thin-Layer Chromatography (TLC)

Both neutral and polar lipids were subjected to one-dimensional thin-layer chromatography (TLC) for lipid class separation and identification, using TLC plates coated with silica gel 60 (Silica Gel 60, 20 × 20 cm, 0.25 mm thickness, Merck, Darmstadt, Germany). Neutral lipids were eluted using petroleum ether:diethyl ether:acetic acid 80:20:1 (*v*/*v*/*v*). Polar lipids were eluted into individual lipids using a five-component mixture of chloroform:acetone:methanol:acetic acid:water 50:20:10:10:5 (*v*/*v*).

Two-dimensional TLC was later performed to confirm the tentative identification of polar lipid classes, using chloroform/methanol/water 65:25:4 (*v*/*v*) as first solvent, and a mixture of chloroform/acetone/methanol/acetic acid/water 50:20:10:10:4 (*v*/*v*) as second solvent.

A brief exposure to iodine vapor was used as general staining to visualize the different lipid spots subsequently recovered from the TLC plates. Each individual class was then subjected to gas chromatography (GC) for quantification of its fatty acid profile, after direct-transmethylation.

Individual lipid classes were identified by comparison of *R*_f_ values to *R*_f_ values provided in the literature [70], by using specific lipid charring reagents as described by Mereiles *et al.* [46], and/or by running commercial lipid standards (Avanti^℘^ Polar Lipids, Inc., Alabaster, AL, USA) along with the samples.

### 4.6. Fatty Acid Analysis

Fatty acid methyl esters (FAME) were obtained by transmethylation of an aliquot of total lipid extract, or by direct-transmethylation of the silica powder containing the different lipid class fractions and/or the freeze-dried algal powder, with dry methanol containing 2% (*v*/*v*) H_2_SO_4_ and heating at 80 °C for 1.5 h with continuous stirring under a nitrogen atmosphere as described by Schmid *et al.* [26].

Gas chromatographic analysis of FAME was performed on an Agilent 7890A GC (Agilent Technologies) equipped with a flame ionization detector and a fused silica capillary column (DB-WAXETR, 0.25 mm × 30 m × 0.25 µm, Agilent Technologies, Santa Clara, CA, USA). Hydrogen was used as a carrier gas. The injector and detector temperatures were 250 °C and 300 °C, respectively. The temperature was programmed at 140 °C for 1 min, raised from 140 to 200 °C at a rate of 15 °C·min^−1^, and then from 200 to 250 °C at a rate of 2 °C·min^−1^.

Identification of FAME was obtained by co-chromatography with authentic commercially available FAME standards (Supelco™ 37 Component FAME Mix, Supelco, Bellefonte, PA, USA) and FAME of fish oil (Menhaden Oil, Supelco). Fatty acid contents were quantified by comparison with a known amount of added pentadecanoic acid 15:0 (Pentadecanoic acid, 99%, Catalog No. A14664-09, Alfa Aesar, Heysham, England, UK) as internal standard. Results are expressed as the mean values of two replicates (*n* = 2) obtained from two analytical repetitions for each extract.

### 4.7. Pigment Analysis

Pigments were analyzed according to the method described by Wright *et al.* [72], modified by Bidigare *et al.* [73]. A known amount of total crude lipid extract was re-suspended in a known volume of cold 90% aqueous acetone (HPLC grade, Fischer Scientific, Loughborough LE11 5RG, England, UK). After overnight at 4 °C to allow full re-dissolution of algal pigments, the samples were filtered before High Performance Liquid Chromatography (HPLC) analysis.

Pigments were separated and quantified on a Agilent 1200 series HPLC (Agilent Technologies) equipped with a Diode Array Detector (DAD) and a Fluorescence Detector (FLD), and separated using a C18 column (150 mm × 4.6 mm inner diameter, Eclipse XDB-C18, Agilent Technologies). The gradient mobile phase consisted of 80:20 (*v*/*v*) methanol/0.5 M ammonium acetate (pH = 7.2, with 0.1% (*w*/*v*) added butylated hydroxytoluene (BHT)), 87.5:12.5 (*v*/*v*) acetonitrile/Milli-Q water (0.1% (*w*/*v*) added BHT) and ethyl-acetate. Identification of algal pigments was obtained by comparison of peak retention times and spectra with commercial standards (DHI, Hørsholm, Denmark; Sigma-Aldrich, St. Louis, MO, USA). Pigment concentrations were calculated using standard curves obtained by injection of precisely quantified amounts of commercial pigment standards. Results are expressed as the mean values of two replicates (*n* = 2) obtained from two analytical repetitions for each extract.

### 4.8. Cell Culture

Human THP-1 monocytes were cultured in RPMI 1640 medium supplemented with 10% (*v*/*v*) heat inactivated fetal bovine serum (FBS), L-glutamine and 0.5% (*w*/*v*) penicillin/streptomycin, and incubated at 37 °C in a 5% CO_2_ atmosphere. For monocyte-macrophage differentiation, cells were seeded at a density of 2 × 10^5^ cell·mL^−1^ in 12-well plates or 5 × 10^4^ cell·mL^−1^ in 96-well plates, and differentiation was induced by exposing the cells to 10 ng·mL^−1^ phorbol-12-myristate-13-acetate (PMA) in 2.5% (*v*/*v*) FBS supplemented media for 72 h. Macrophages were subsequently treated with PMA-free media with 2.5% FBS for a further 24 h. Lipid extracts from *P. lutheri*, *P. palmata*, *P. dioica* and *C. crispus* were prepared in dimethyl sulfoxide (DMSO) and added to fresh culture medium at a concentration of 3 µg·mL^−1^ total fatty acids and incubated for 24 h. Control cells were treated with DMSO alone at a concentration of 0.1% (*v*/*v*).

### 4.9. Cytotoxicity Assay

Cytotoxicity of the extracts was measured by MTS (3-(4,5-dimethylthiazol-2-yl)-5-(3-carboxymethoxyphenyl)-2-(4-sulfophenyl)-2H-tetrazolium) assay using the CellTiter 96^®^ AQ_ueous_ One Solution Assay (Promega Corporation, Madison, WI, USA) according to manufacturer’s instructions. Briefly, cells were seeded in a 96-well plate for 24 h and then treated with the algal lipid extracts at different concentrations. Subsequently, 20 µL of MTS reagent was added into each well. Following incubation at 37 °C and 5% CO_2_ for 4 h, the absorbance at 490 nm with reference wavelength of 610 nm was read using a microplate reader. For subsequent analysis, cells were treated with a concentration of extract that exhibited no significant reduction in cell viability.

### 4.10. Cytokine Analysis

For cytokine analysis, differentiated THP-1 macrophage cells were first pretreated with algal lipid extracts for 24 h followed by induction of pro-inflammatory response by treating with 0.1 µg·mL^−1^ LPS for 24 h. Following the LPS stimulation, cell culture supernatants were collected, centrifuged at 1000× *g* for 10 min and stored at −80 °C until further analysis. Abundance of cytokine (TNFα, IL-6, IL-8, IL-1β, IL-12p70, IFNγ, IL-10) in the cell culture supernatants was performed using the MSD Human proinflammatory 7-plex Ultra-sensitive electrochemiluminescent (ECL) assay (Meso Scale Discovery (MSD), Gaithersburg, MD, USA). Briefly, 25 µL of supernatant samples were added to wells of a 96-well plate precoated with primary capture antibodies. The plate was then sealed and incubated for 1 h at room temperature with vigorous shaking (300 rpm). Detection antibody (25 µL) was then added to the wells and the plate was sealed and incubated for 1 h at room temperature with continuous shaking (300 rpm). The plate was then washed three times with phosphate-buffered saline (PBS) containing 0.05% Tween-20 prior to the addition of 150 µL read buffer. The plate was then read in a SECTOR Imager (Meso Scale Discovery) and quantification of individual cytokines was performed following the supplier’s guidelines.

### 4.11. Preparation of RNA and cDNA Synthesis

Total RNA was extracted from THP-1 macrophages using a Qiagen RNeasy Mini Kit (Qiagen, Manchester, UK) according to the manufacturer’s protocol. RNA was dissolved in 20 μL of nuclease-free water and then subjected to deoxyribonuclease I (DNase I) treatment to eliminate the genomic DNA contamination. RNA yield was assessed by measuring absorbance at 260 nm using a Nano-drop ND-1000 spectrophotometer (Thermo Fischer Scientific, Waltham, MA, USA). Sufficient RNA purity was determined by a 260:280 nm absorbance ratio of ≥1.8 and by visualizing on a 1.5% (*w*/*v*) RNA agarose gel. Total RNA (1 µg) was reverse-transcribed to cDNA using a RevertAid H Minus First Strand cDNA synthesis Kit (Fermentas GmbH, St. Leon-Rot, Germany) according to the manufacturer’s protocol.

### 4.12. Quantitative Real-Time PCR Analysis

The quantitative expression of a panel of 96 genes (list of genes provided in supplementary Table S1) involved in the inflammatory signaling cascade in human was evaluated using a PCR array in a LightCycler 480 instrument (Roche Diagnostics GmbH, Mannheim, Germany). This is a customized array designed by Sigma-Aldrich Corp. that enables screening of 92 different genes involve in inflammatory signaling pathways and 4 internal reference genes in human. For the PCR array experiment, 25 µL cDNA (after 1:5 dilution) from each individual wells within a single treatment group were pooled. Real-time reverse transcriptase PCR was performed on a 20 µL reaction mixture per well containing 1 µL pooled cDNA, 9 µL water and 10 µL SYBR green master mix (Applied Biosystems, Foster City, CA, USA). The thermal cycle conditions were 94 °C for 30 s followed by 60 °C for 1 min, for 40 cycles. The mRNA abundances were expressed in crossing point (Cp) values, the number of PCR cycles after which the PCR product crosses a threshold value. In this experiment, a Cp value of 35 was considered as the cut-off limit.

Normalization of the expression of 92 target genes included in the PCR array was performed based on three reference genes (beta actin (*ACTB*), glyceraldehyde 3-phosphate dehydrogenase (*GAPDH*) and beta glucuronidase (*GUSB*)) and following 2^−ΔΔCp^ method. Briefly, average ΔCp was calculated as the difference of Cp values of any target gene minus average of the Cp value of the three reference genes. Then, fold change was calculated as 2^(−average ΔCp target gene)^/2^(−average ΔCp reference gene)^. A fold difference cut-off point was set at ≥2.5.

Validation of the PCR array data was performed through evaluation of the quantitative expression of three target genes (*TLR8*, *TLR1* and *TRAF5*) and one reference gene (*GAPDH*) in each of the replicate samples on the LightCycler 480 instrument (Roche Diagnostics GmbH). The PCR reaction (10 µL) contained 1 µL cDNA (following 1:5 dilution), 3 µL nuclease free water, 1 µL forward and reverse primer and 5 µL SYBR green master mix (Roche Diagnostics GmbH). The thermal cycle conditions were as follows: 94 °C for 10 min followed by 40 cycles of 94 °C for 30 s, 60 °C for 30 s, and 72 °C for 30 s. Samples were analyzed in triplicate and Cp values <35 were used for analysis. The mRNA abundance of three target genes (*TLR8*, *TLR1* and *TRAF5*) were normalized against the expression of *GAPDH*.

### 4.13. Statistical Analysis

Statistical analysis was performed using GraphPad Prism version 6.0 for Windows (GraphPad Software). Extract composition results are expressed as the mean values of two replicates (*n* = 2) ± standard deviation (SD) obtained from two analytical repetitions for each extract. Cytokine production and qPCR results are presented as means ± standard error of the mean (SEM) of at least three independent experiments. To assess whether differences between the exposures were significant, multiple comparisons were performed using one-way analysis of variance (ANOVA) followed by Dunnett’s Multiple Comparison Test or Fisher’s least significant difference test. A *p*-value of ≤0.05 was considered statistically significant.

## Supplementary Files

Supplementary File 1
