# Supplementary material for: The Anti-Inflammatory Effect of Algae-Derived Lipid Extracts on Lipopolysaccharide (LPS)-Stimulated Human THP-1 Macrophages
Source: Mar Drugs. 2015 Aug 20;13(8):5402–24. doi: 10.3390/md13085402 (PMC4557028; doi:10.3390/md13085402)
Supplement: Supplementary File 1 [file marinedrugs-13-05402-s001.docx]

**Supplementary Materials**

**Table S1.** Symbols and names of 96 inflammation-associated genes screened in gene expression array.

| **Gene symbol** | **Gene name** |
| --- | --- |
| *ACTB* | beta actin |
| *AKT1* | v-akt murine thymoma viral oncogene homolog 1 |
| *C3* | complement component 3 |
| *CCL15* | chemokine (C-C motif) ligand 15 |
| *CCL16* | chemokine (C-C motif) ligand 16 |
| *CCL17* | chemokine (C-C motif) ligand 17 |
| *CCL20* | chemokine (C-C motif) ligand 20 |
| *CCL25* | chemokine (C-C motif) ligand 25 |
| *CCR1* | chemokine (C-C motif) receptor 1 |
| *CCR4* | chemokine (C-C motif) receptor 4 |
| *CCR5* | chemokine (C-C motif) receptor 5 |
| *CCR6* | chemokine (C-C motif) receptor 6 |
| *CCR8* | chemokine (C-C motif) receptor 8 |
| *CD14* | CD14 molecule |
| *CD40* | CD40 molecule, TNF receptor superfamily member 5 |
| *CD83* | CD83 molecule |
| *CLEC7A* | C-type lectin domain family 7, member A |
| *CREB1* | cAMP responsive element binding protein 1 |
| *CRP* | C-reactive protein, pentraxin-related |
| *CYSLTR1* | cysteinyl leukotriene receptor 1 |
| *GAPDH* | glyceraldehyde-3-phosphate dehydrogenase |
| *GUSB* | glucuronidase, beta |
| *HPRT1* | hypoxanthine phosphoribosyltransferase 1 |
| *HRH3* | histamine receptor H3 |
| *ICAM1* | intercellular adhesion molecule 1 |
| *IFNG* | interferon gamma |
| *IKBKB* | inhibitor of kappa light polypeptide gene enhancer in B-cells, kinase beta |
| *IL10* | interleukin 10 |
| *IL12B* | interleukin 12b |
| *IL17A* | interleukin 17A |
| *IL1B* | interleukin 1, beta |
| *IL1R1* | interleukin 1 receptor, type I |
| *IL1RN* | interleukin 1 receptor antagonist |
| *IL2* | interleukin 2 |
| *IL23* | interleukin 23 |
| *IL2RA* | interleukin 2 receptor, alpha |
| *IL4* | interleukin 4 |
| *IL6* | interleukin 6 |
| *IL6R* | interleukin 6 receptor |
| *IL6ST* | interleukin 6 signal transducer |
| *IL8* | interleukin 8 |
| *INSR* | insulin receptor |
| *JAK2* | Janus kinase 2 |
| *LTA* | lymphotoxin alpha |

**Table S1.** *Cont.*

| *LTB4R2* | leukotriene B4 receptor 2 |
| --- | --- |
| *LTC4S* | leukotriene C4 synthase |
| *MALT1* | mucosa associated lymphoid tissue lymphoma translocation gene 1 |
| *MAP3K1* | mitogen-activated protein kinase kinase kinase 1 |
| *MAPK1* | mitogen-activated protein kinase 1 |
| *MAPK8* | mitogen-activated protein kinase 8 |
| *MMP1* | matrix metallopeptidase 1 (interstitial collagenase) |
| *MMP13* | matrix metallopeptidase 13 (collagenase 3) |
| *MMP9* | matrix metallopeptidase 9 (gelatinase B, 92 kDa gelatinase, 92 kDa type IV collagenase) |
| *MTHFR* | methylenetetrahydrofolate reductase (NAD(P)H) |
| *MYD88* | myeloid differentiation primary response gene 88 |
| *NFKB1* | nuclear factor of kappa light polypeptide gene enhancer in B-cells 1 |
| *NFKB1A* | nuclear factor kappa B 1 A |
| *NFKB2* | nuclear factor of kappa light polypeptide gene enhancer in B-cells 2 (p49/p100) |
| *NOS2* | nitric oxide synthase 2, inducible |
| *NR2C2* | nuclear receptor subfamily 2, group C, member 2 |
| *NR3C1* | nuclear receptor subfamily 3, group C, member 1 (glucocorticoid receptor) |
| *NR4A1* | nuclear receptor subfamily 4, group A, member 1 |
| *NR4A2* | nuclear receptor subfamily 4, group A, member 2 |
| *PLA2G2D* | phospholipase A2, group IID |
| *PPARG* | peroxisome proliferator-activated receptor gamma |
| *PTGER1* | prostaglandin E receptor 1 (subtype EP1) |
| *PTGER2* | prostaglandin E receptor 2 (subtype EP2) |
| *PTGS2* | prostaglandin-endoperoxide synthase 2 (prostaglandin G/H synthase and cyclooxygenase) |
| *REL* | v-rel avian reticuloendotheliosis viral oncogene homolog |
| *RELA* | v-rel avian reticuloendotheliosis viral oncogene homolog A |
| *RIPK1* | receptor (TNFRSF)-interacting serine-threonine kinase 1 |
| *SOCS3* | suppressor of cytokine signaling 3 |
| *SELE* | selectin E |
| *SOCS1* | suppressor of cytokine signaling 1 |
| *SOX9* | SRY (sex determining region Y)-box 9 |
| *STAT3* | signal transducer and activator of transcription 3 (acute-phase response factor) |
| *TGFB1* | transforming growth factor, beta 1 |
| *TIRAP* | toll-interleukin 1 receptor (TIR) domain containing adaptor protein |
| *TLR1* | toll-like receptor 1 |
| *TLR2* | toll-like receptor 2 |
| *TLR3* | toll-like receptor 3 |
| *TLR4* | toll-like receptor 4 |
| *TLR6* | toll-like receptor 6 |
| *TLR7* | toll-like receptor 7 |
| *TLR8* | toll-like receptor 8 |
| *TNFA* | tumor necrosis factor a (TNF superfamily, member 2) |
| *TNFRSF10B* | tumor necrosis factor receptor superfamily, member 10b |
| *TNFRSF1A* | tumor necrosis factor receptor superfamily, member 1A |
| *TNFSF18* | tumor necrosis factor (ligand) superfamily, member 18 |
| *TP53* | tumor protein p53 |
| *TRAF1* | TNF receptor-associated factor 1 |
| *TRAF2* | TNF receptor-associated factor 2 |

**Table S1.** *Cont.*

| *TRAF3* | TNF receptor-associated factor 3 |
| --- | --- |
| *TRAF5* | TNF receptor-associated factor 5 |
| *TRAF6* | TNF receptor-associated factor 6 |
| *VCAM* | vascular cell adhesion molecule |

© 2015 by the authors; licensee MDPI, Basel, Switzerland. This article is an open access article distributed under the terms and conditions of the Creative Commons Attribution license (http://creativecommons.org/licenses/by/4.0/).
